# Efficacy and complications of endoscopic thoracoscopic versus laparoscopic radical esophagectomy in esophageal cancer treatment: A retrospective study

**DOI:** 10.1097/MD.0000000000038645

**Published:** 2024-09-06

**Authors:** Mingquan Ma, Peng Ren, Haitong Wang, Hongdian Zhang, Lei Gong, Yufeng Qiao, Xiangming Liu, Peng Tang

**Affiliations:** a Department of Esophageal Cancer, Tianjin Medical University Cancer Institute and Hospital, Key Laboratory of Cancer Prevention and Therapy of Tianjin, Tianjin’s Clinical Research Center for Cancer, National Clinical Research Center of Cancer, Hexi, Tianjin, China.

**Keywords:** complications, efficacy, endoscope-thoracoscope and laparoscope, radical esophagectomy

## Abstract

To evaluate the efficacy and postoperative complications of endoscopic thoracoscopic and laparoscopic radical esophagectomy compared to open surgery in esophageal cancer treatment. This retrospective study included 103 esophageal cancer patients admitted from August 2018 to March 2022, divided into observation (endoscopic surgery) and control (open surgery) groups. We compared intraoperative parameters, postoperative outcomes, immune function, and one-year overall survival (OS). Intraoperative bleeding volume, the retention time of chest tube, postoperative fasting time, and hospital stay in the observation group were smaller than those in the control group (*P* < .05). The differences were not statistically significant (*P* > .05) when comparing operative time, the number of intraoperative blood transfusion cases, and the rate of operating room extubation in these 2 groups. The differences were not statistically significant when comparing the amount of resected lymph nodes and the positive rate of incisal edge in these 2 groups (*P* > .05). There was no statistically significant difference in the complication rates such as pneumonia, pleural effusion, pneumothorax, pulmonary embolism, anastomotic fistula, the leakage of thoracic duct, the injury of RLN and arrhythmia in these 2 groups (*P* > .05). At 7 days postoperatively, the CD4+ and CD4+/CD8+ in the observation group and the control group were smaller than the preoperative ones in their same groups, and they were larger in the observation group than those in the control group (*P* < .05); There was no statistically significant difference on the CD8+ in the observation group and the control group at 7 days postoperatively compared with the preoperative ones in their same groups (*P* > .05). The 1-year postoperative OS rate was 81.63% (40/49) in the observation group and 72.22% (39/54) in the control group, and the difference was not statistically significant when comparing the OS rates of these 2 groups (*P* = .238, HR = 0.622, 95% CI = 0.279–1.385). Endoscopic thoracoscopic and laparoscopic esophagectomy offers less invasive treatment with significant short-term benefits and better preservation of immune function in esophageal cancer patients, making it a safe and effective surgical option.

## 1. Introduction

Esophageal cancer is one of the common malignancies in the digestive system. A global and Chinese cancer disease burden in 2020 statistics show^[1]^ that from 2015 to 2020 the number of new cases of esophageal cancer in China increases from 246,000 to 324,000, and the death toll increases from 188,000 to 301,000. China is a region with high incidence of esophageal cancer, and its incidence and mortality rate are on the rise year by year. Early and mid-stage esophageal cancer patients are mainly treated by surgical resection, and chemotherapy and radiotherapy are supplementary treatments, while advanced esophageal cancer patients are mainly treated by chemotherapy and radiotherapy, and surgical resection is supplementary treatment.^[2,3]^ And after advanced patients receive neoadjuvant or chemotherapy and radiotherapy, surgery seems to be their only choice to cure esophageal cancer.^[4,5]^ However, despite recent improvements in diagnosis, surgical treatment and neoadjuvant, the prognosis for patients with esophageal cancer remains poor, with an overall 5-year survival rate of only 5% to 15%.^[6]^ This may be related to the shortcomings of conventional open radical esophagectomy. Although traditional open radical esophagectomy can effectively remove lymph nodes, long-term clinical practice has found that it has significant surgical damage, many postoperative complications, significant decrease in patient immune function, and poor prognosis.^[7,8]^ For example, some studies have shown that the incidence of postoperative morbidity, especially pulmonary complications, in patients with esophageal cancer ranges from 30% to 50%, while the postoperative mortality ranges from 2% to 10%.^[9]^ Therefore, it is important to explore the importance of reducing surgical risks and decreasing the incidence of intraoperative and postoperative complications and perioperative mortality in esophageal cancer.

With the advancement of endoscope-thoracoscope and laparoscope technology, the minimally invasive esophagectomy for esophageal cancer is getting growing attention from thoracic surgeons. At present, the commonly used surgical methods of minimally invasive esophagectomy include endoscopic mucosal resection, thoracoscopic esophagectomy, mediastinoscopic esophagectomy, thoraco-laparoscopic esophagectomy, and various endoscopes can be used alone or in combination with each other to treat esophageal cancer. Endoscope-thoracoscope and laparoscope has the advantages of minimally invasive, small traumatic surface, less bleeding, less postoperative complications, fast recovery and the high quality of short-term life,^[10,11]^ which has been paid growing attention by the medical profession. It has provided new ideas and development direction for the surgical treatment of esophageal cancer. At present, the technique of thoracic-laparoscopic radical resection on esophageal cancer (also called endoscope-thoracoscope and laparoscope radical esophagectomy) has been developed rapidly and has become a mature technique for clinical application.^[12]^ Based on this, the study objective of this research was to analyze the endoscope-thoracoscope and laparoscope radical resection to investigate its short-term and long-term efficacy in the treatment of elderly patients with esophageal cancer and to analyze the incidence of postoperative complications of this treatment option.

## 2. Methodologies

### 2.1. Research subjects

The study was approved by the Ethics Committee of Tianjin Medical University Cancer Institute and Hospital, and the patients and their families signed the consent form. In this retrospective trial, we randomly selected 103 cases of patients with esophageal cancer admitted to our hospital from August 2018 to March 2022 as study subjects for this research. The inclusion criteria for this study were as follows: (1) Complete clinical data. (2) The diagnosis was confirmed through gastroscopy biopsy, upper gastrointestinal barium meal examination, etc, and the length of the lesion was ≤7 cm. (3) Preoperative chest enhanced CT and endoscopic ultrasound indicate no significant external invasion of the tumor. (4) The transverse diameter of the tumor is ≤3 cm, and the maximum diameter of enlarged lymph nodes in the mediastinum is ≤2 cm. (5) Preoperative chest X-ray, electrocardiogram, lung function, chest CT, and other examinations showed no liver lung metastasis, no cervical lymph node metastasis, and no invasion of surrounding organs. The heart and lung function can withstand surgery. (6) The patient has provided informed consent for the treatment. The exclusion criteria for this study were as follows: (1) preoperative chemotherapy, radiotherapy, and biologically targeted therapy. (2) Previous history of chest and abdominal trauma or major surgery. (3) Patients who undergo emergency surgery due to perforation and massive bleeding. A total of 103 cases were enrolled in the study group. According to different treatment methods, patients who underwent thoracoscopic esophagectomy for esophageal cancer were included in the observation group, while patients who underwent open esophagectomy were included in the control group.

### 2.2. Method

#### 2.2.1. Observation group

Double-lumen tracheal intubation and general anesthesia were performed under the routine general anesthesia. The patient was placed in a semi-prone position on the left side, and a 1 cm incision was made at the 7th intercostal space in the right mid-axillary line to place the thoracoscope as an observation hole. A 0.5 cm incision was made in the 3rd intercostal space in the anterior axillary line and the 7th and 9th intercostal spaces in the subscapular line as the operation hole. Thoracoscopic exploration was performed to understand whether the thoracic cavity was adherent, and whether there was obvious invasion on the size and location of the tumor. The specific location of the operation hole was decided according to the location and anatomical characteristics of the tumor in the patient’s chest cavity, and there was no fixed location. The upper mediastinal pleura is opened first, and the right recurrent laryngeal nerve (RLN) is carefully exposed and the lymph nodes adjacent to the right RLN are cleared. Then azygos arch was freed, and the whole circumference of the esophagus was freed from top to bottom, taking care to avoid damaging the thoracic duct during the freeing process. The intrinsic esophageal artery was dissected by an ultrasonic knife, and the azygos arch was clamped and cut by a titanium clip. The paraesophageal lymph node was also removed. The submental lymph nodes of protuberance and the lymph nodes adjacent to the left RLN were cleared. After checking that there was no bleeding and no air leak, one chest drain and one mediastinal drain were left in place. The chest incision was closed. The patient was placed in a lying head-high, foot-low position with the head tilted to the right. A 4 cm incision was made in front of the left sternocleidomastoid muscle to separate the esophagus, and the anastomosis was reserved and the esophageal stump was closed with a distal suture. A 1cm incision was made at the left edge of the umbilicus, and a pneumoperitoneum needle was placed to form an artificial pneumoperitoneum, and the intra-abdominal pressure was maintained at about 15 mm Hg, and a laparoscope was placed. The abdominal operating holes were located 2 cm above the umbilicus in the midclavicular line, 2 cm below the rib margin in the right midclavicular line, and below the xiphoid process, respectively. The lateral omentum of the large and small curves of the stomach was freed, the left artery of the gastric omentum, the peripheral vascular of gastric cardia, and the gastrocolic ligament were dissected, the left gastric artery was clamped and dissected, the stomach was freed, and the lymph nodes in the left gastric, common hepatic arteries and the adjacent part celiac artery were cleared. The incision of inferior xiphoid process was extended to 3 to 4 cm, and the stomach was cut into a tube shape with a linear Endo-GIA Stapler, and the large curved tube was lifted along the original esophageal bed to the neck with a connecting wire, and the fundus of stomach was cut, and an PPH was placed, and the esophagus was in the anastomosis with the large-curved lateral wall instruments of stomach body. The fundus of stomach was partially excised and reinforced with sutures using an Endo-GIA Stapler. A film drainage was left in place and the neck incision was sutured. The abdominal cavity was checked again for no bleeding, one abdominal drain was left in place, and the abdominal incision was closed layer by layer.

The indications for thoracolaparoscopic esophageal cancer radical surgery are as follows: individuals diagnosed with early-stage esophageal cancer, as well as some third-stage lower esophageal cancer, with a tumor length of <5 cm, and in generally good condition. Patients should have no distant metastasis, no serious damage to heart, lung, liver, kidney function, or any other surgical contraindications. They should actively consider surgical treatment. However, for individuals over 70 years old, a strict selection process should be followed.

#### 2.2.2. Control group

Conventional radical esophagectomy was performed under general anesthesia with tracheal intubation. The patient was placed in the left lateral position, prepared by routine towel disinfection, and an incision of about 15–20 cm in length was made on the right lateral chest to enter the thoracic cavity and free the thoracic esophagus and clear the lymph nodes. Then, the position was changed to supine, and an incision of about 10–15 cm in length was made in the abdomen, and the stomach was freed after entering the abdomen from the xiphoid process to the umbilicus, and the lymph nodes were cleared. Finally, a 5 cm-long incision was made on the medial side of the left sternocleidomastoid muscle to free the cervical esophagus, remove the cervical lymph nodes, and create a tubular stomach followed by anastomosis of the stomach and esophagus. After the operation, a drainage tube was placed after flushing with sterile water, and the incision was sutured. Postoperatively, vital signs were routinely monitored, analgesic pumps were used for analgesia, and routine anti-infective treatment was performed.

The indications for open radical surgery for esophageal cancer include the following criteria: the patient must be in good overall condition, with no apparent abnormalities in cardiopulmonary function, and must be able to tolerate surgical treatment prior to undergoing radical surgery. The patient should not have coagulation dysfunction, or if they do, it should be treated before surgery. Esophageal cancer patients with clinical staging of stage 1, stage 2, and some stage 3 are eligible for this procedure. Patients who experience recurrence after local radiotherapy but do not have distant organ metastasis, and whose physical condition permits surgical treatment, can also undergo the surgery. In cases of squamous cell carcinoma or cervical esophageal cancer with longer lesions, complete resection may not be feasible. However, if the patient is in good overall condition, preoperative neoadjuvant radiotherapy and chemotherapy can be administered to shrink the tumor before surgical treatment.

### 2.3. Evaluation of clinical efficacy

Clinical outcomes were assessed in terms of surgical outcome, oncologic outcome, and perioperative complications. Surgical outcomes included: Operation time, intraoperative bleeding volume, the number of intraoperative blood transfusion cases, postoperative operating room extubation rate, thoracic duct retention time, postoperative fasting time and hospital stay; Oncological outcomes included: the amount of lymph nodes resection, positive margin rate; Perioperative complications were mainly observed for the incidence of complications such as respiratory complications (including pneumonia, pleural effusion, pneumothorax, pulmonary embolism) and arrhythmias, anastomotic fistula, the leakage of thoracic duct and the injury of RLN.

### 2.4. Immune function

To compare the levels of peripheral blood T-lymphocyte subsets (CD4+, CD8+, and CD4+/CD8+) in these 2 groups before and 7 days after surgery: 3 mL of peripheral elbow venous blood was collected from patients in the early morning before and 7 days after surgery, respectively, anticoagulated with heparin, and the levels of CD4+ and CD8+ were detected by FACSCalibur flow cytometer and its corollary reagents (BD), and counted the CD4+/CD8+.

### 2.5. Postoperative follow-up

Postoperative follow-ups were conducted by dedicated staff via outpatient clinics, door-to-door visits, sending text messages or making phone calls. Follow-up visits were conducted once a month lasted for 1 year. Patients underwent routine physical examination, laboratory tests, and chest and abdominal CT. The overall survival (OS) of the 2 groups was counted. The endpoint of OS was death or lost to follow-up due to any cause.

### 2.6. Statistical analysis

Data analysis was performed by SPSS23.0. Continuous variables such as age, operation time, intraoperative bleeding volume, thoracic duct retention time, postoperative fasting time, hospital stay, ICU monitoring time, the amount of lymph nodes resection, CD4+, CD8+, and CD4+/CD8+ were tested for normality to confirm that they conformed to a normal distribution and were expressed as mean ± standard deviation, and significant differences between these 2 groups were assessed by independent sample *t* test, and paired sample *t* test before and after treatment within the groups. Categorical variables such as gender, tumor site, TNM stage, the number of intraoperative transfusions cases, postoperative operating room extubation rate, positive margin rate, and complication rate were expressed as percentages, and Pearson chi-square test, Fisher exact test, or Mann–Whitney test were used to assess significant differences between groups. Kaplan–Meier curves were plotted by GraphPad Prism and were used to analyze the OS and DFS of these 2 groups. The log-rank test was used to compare the OS between these 2 groups. *P* < .05 indicating a statistically significant difference.

## 3. Results

### 3.1. Comparison of general information

Compared with the control group, there was no statistically significant difference in the general data of age, gender, tumor site and TNM stage in the observation group (*P* > .05). See Table 1.

**Table 1:** Comparison of general information [n (%), x ± s].

Projects	Observation group (n = 49)	Control group (n = 54)	*t*/*X*²	*P*
Age (years)	62.95 ± 6.53	61.28 ± 7.79	1.171	.244
*Gender*			0.016	.898
Male	33 (67.35%)	37 (68.52%)		
Female	16 (32.65%)	17 (31.48%)		
*Tumor site*			0.520	.602
Upper section	5 (10.20%)	6 (11.11%)		
Middle section	30 (61.23%)	29 (53.70%)		
Lower section	14 (28.57%)	19 (35.19%)		
*TNM staging*			0.406	.684
Period I	13 (26.53%)	12 (22.22%)		
Period II	24 (48.98%)	28 (51.85%)		
Period III	12 (24.49%)	14 (25.93%)		

### 3.2. Comparison of surgical outcomes

The intraoperative bleeding, the retention time of thoracic duct, postoperative fasting time, and hospital stay in the observation group were smaller than those in the control group (*P* < .05); There were no statistically significant differences between the observation group and the control group in terms of operating time, number of intraoperative blood transfusion cases, and operating room extubation rate (*P* > .05). See Table 2.

**Table 2:** Comparison of surgical outcomes [n (%), x ± s].

Projects	Observation group (n = 49)	Control group (n = 54)	*t*/*X*²	*P*
Surgery time (minutes)	272.65 ± 19.92	266.33 ± 21.58	1.537	.127
Intraoperative bleeding volume (mL)	152.81 ± 29.23	214.55 ± 38.06	9.162	**<.001**
Number of intraoperative blood transfusions cases	3 (6.12%)	5 (9.26%)	0.050	.821
Operating room extubation rate	48 (97.96%)	51 (94.44%)	0.850	.356
Duration of chest tube retention (day)	9.51 ± 4.57	13.55 ± 4.25	4.658	**<.001**
Duration of postoperative fasting (day)	9.66 ± 2.51	12.38 ± 4.80	3.564	**<.001**
Hospital stay (day)	15.48 ± 4.11	18.54 ± 5.12	3.325	**.001**

### 3.3. Comparison of oncology outcomes

There was no statistically significant difference in the amount of lymph nodes resection and the positive margin rate between the observation group and the control group (*P* > .05). See Table 3.

**Table 3:** Comparison of oncological outcomes [n (%)].

Group	n	Amount of lymph nodes resection (pcs)	Positive rate of cutting edge
Observation group	49	17.72 ± 3.34	0 (0.00%)
Control group	54	18.92 ± 3.43	2 (3.70%)
*t*/*X*²		1.790	0.416
*P*		.076	.518

### 3.4. Comparison on the incidence of complications

**Figure 1.**
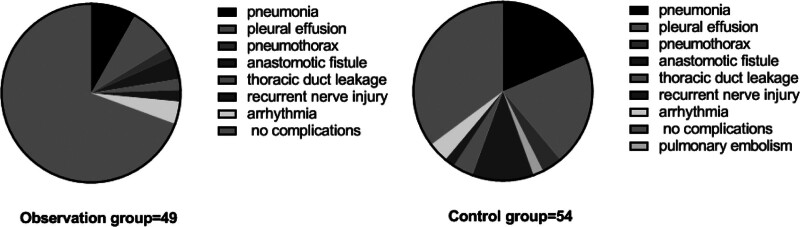
Comparison of complication rates in 2 groups.

There was no statistically significant difference (*P* > .05) between the incidence of complications such as pneumonia, pleural effusion, pneumothorax, pulmonary embolism, anastomotic fistula, thoracic duct leakage, RLN injury, and arrhythmia between the observation group and the control group. See Table 4 and Figure 1.

**Table 4:** Comparison of complication rates [n (%)].

Projects	Observation group (n = 49)	Control group (n = 54)	*X*²	*P*
Pneumonia	4 (8.16%)	10 (18.51%)	2.345	.125
Pleural effusion	4 (8.16%)	11 (20.37%)	3.076	.079
Pneumothorax	1 (2.04%)	2 (3.70%)	0.007	.931
Pulmonary embolism	0 (0.00%)	1 (1.85%)	0.002	.961
Anastomotic fistula	2 (4.08%)	6 (11.11%)	0.926	.335
Thoracic duct leakage	1 (2.04%)	2 (3.70%)	0.007	.931
Recurrent laryngeal nerve injury	1 (2.04%)	1 (1.85%)	0.416	.518
Arrhythmia	2 (4.08%)	2 (3.70%)	0.169	.680

### 3.5. Comparison of immune function

**Figure 2.**
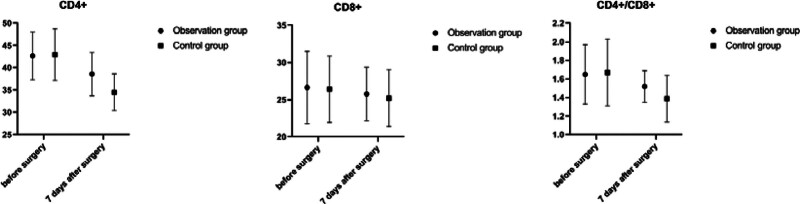
Comparison of immune function between 2 groups.

Preoperatively, there were no statistically significant differences in the CD4+, CD8+, and CD4+/CD8+ of the observation group and the control group (*P* > .05); 7 days after surgery, the CD4+ and CD4+/CD8+ of the observation group and the control group were smaller than those of their same groups before surgery, and those of the observation group was larger than those of the control group (*P* < .05); 7 days after surgery, the CD8+ of the observation group and the control group compared with that of their same groups before surgery, and the difference was not statistically significant (*P* > .05). The difference was not statistically significant when comparing the CD8+ in the observation group and the control group with that in their preoperative periods (*P* > .05). See Table 5 and Figure 2.

**Table 5:** Comparison of immune function (x ± s).

Projects	Observation group (n = 49)	Control group (n = 54)	*t*	*P*
CD4+ (%)				
*Preoperative*	42.67 ± 5.33	42.99 ± 5.81	0.290	.772
7 days after surgery	38.63 ± 4.87*	34.54 ± 4.12*	4.608	**<.001**
CD8+ (%)				
*Pre-operative*	26.67 ± 4.87	26.44 ± 4.46	0.254	.800
7 days after surgery	25.78 ± 3.58	25.22 ± 3.81	0.758	.450
CD4+/CD8+				
*Pre-operative*	1.65 ± 0.32	1.67 ± 0.36	0.256	.798
7 days after surgery	1.52 ± 0.17*	1.39 ± 0.25*	2.993	**.003**

*Note*: Compared with the preoperative situation.

* *P* < .05.

### 3.6. Long-term efficacy analysis

Two groups were followed up for a duration of 3 to 21 months. The endoscopic group consisted of 45 cases, with an average follow-up time of (11.26 ± 3.96) months. In this group, there were 3 cases of recurrence or metastasis, 1 case of death, and an overall survival rate of 97.95%. The control group, on the other hand, had 50 cases and an average follow-up period of (12.19 ± 5.99) months. In this group, there were 4 cases of recurrence or metastasis, 2 deaths, and an overall survival rate of 96.29%. Statistical analysis revealed no significant difference between the 2 groups in terms of the number of follow-up patients, follow-up time, recurrence or metastasis, and overall survival rate. See Table 6.

**Table 6:** Comparison of follow-up results.

Outcomes	Observation group (n = 49)	Control group (n = 54)
Follow up rate n (%)	45 (91.80)	50 (92.60)
Follow-up time t/month	11.26 ± 3.96	12.19 ± 5.99
Recurrence or metastasis n (%)	3 (6.12)	4 (8.00)
Case fatality rate n (%)	1 (2.04)	2 (3.70)
Total survival rate (%)	97.95	96.29

## 4. Discussion

At present, surgery is the main means to treat esophageal cancer. Traditional open radical esophagectomy, as a common clinical procedure for treating esophageal cancer, can improve the survival rate and prolong the survival time of patients to a certain extent, but this procedure is more traumatic with long duration of postoperative pain and more complications, which has a great influence on the surgical efficacy and prognosis.^[13]^ Endoscope-thoracoscope and laparoscope radical resection is a new minimally invasive procedure in recent years, clinically. Compared with traditional open surgery, it has the advantages of small trauma, less bleeding, light postoperative pain, fewer complications, faster recovery and shorter hospital stay, it is currently recognized as a better surgical method for treating esophageal cancer in the world.^[14]^

The duration of surgery and the amount of bleeding volume during surgery are a comprehensive reflections of the surgeon’s surgical proficiency and one of the surgical minimally incisive indicators. The length of surgical incision, postoperative hospital stay, the volume of drainage at postoperative day l, and postoperative thoracic duct retention time are also indicators of the degree of trauma caused to patients by surgery. The minimally invasive nature of endoscope-thoracoscope and laparoscope radical esophagectomy is measured by the operating time, intraoperative bleeding volume, the volume of drainage at postoperative day l, and postoperative hospital stay. The clear surgical field of endoscope-thoracoscope and laparoscope radical resection improves the accuracy of lymph node resection while also reducing the damage to surrounding tissues and organs, indirectly reducing the impact of surgery on patients’ normal postoperative physiological activities such as coughing and sputum, which is conducive to patients’ maintenance of normal postoperative respiratory function and promotes postoperative recovery.^[15]^ The results of this study showed that the intraoperative bleeding volume, thoracic duct retention time, postoperative fasting time and hospital stay in the observation group were smaller than those in the control group (*P* < .05), which was consistent with the literature.^[16]^ The results of this study indicated that there was no statistically significant difference in surgical time between the observation group and the control group. This finding may be attributed to the longer duration of our combined thoracolaparoscopic treatment for esophageal cancer. Thoracolaparoscopic combined therapy is a technically demanding and relatively complex surgery. It necessitates not only extensive experience in traditional esophageal cancer resection but also proficiency in thoracolaparoscopic operations. Consequently, there are challenges in accumulating experience and overcoming the learning curve. From the anatomical point of view, the esophagus is a staged blood supply, with the branch of the inferior thyroid artery supplying blood to the cervical segment of the esophagus, the esophageal branch from the aortic arch of the upper esophagus and the intrinsic artery from the parasternal aorta of the lower esophagus supplying blood to the superior thyroid artery, the common carotid artery, and the thoracic esophagus. While traditional open surgery involves the separation of muscles such as the pectoralis major, the part of latissimus dorsi, serratus anterior and intercostal muscles, the observation group uses thoracoscopy for observation and operation without separating the chest muscles to avoid causing bleeding and trauma. With the local magnification of the thoracoscope, the tissues and blood vessels around the esophagus can be clearly seen, which helps the surgeon to free the relevant nerves and blood vessels and reduce intraoperative bleeding volume. There was no significant difference in the number of lymph nodes clearance between these 2 groups in this study, which indicated that endoscope-thoracoscope and laparoscope radical esophagectomy achieved equal radical results.^[17]^ Moreover, thoracoscopic identification of the RLN and the clearance of surrounding lymph nodes have considerable advantages.^[18]^

A study^[19]^ showed that T-lymphocyte subsets, as the first line of defense against tumors, are closely related to the occurrence and development of esophageal cancer and are also an important factor in postoperative resistance to infection. CD4+ and CD4+/CD8+ ratios directly reflect the cellular immune function of the body. The higher the value, the stronger the immune function of the body.^[20]^ Previous studies^[20,21]^ reported that the autoimmune function of elderly patients with esophageal cancer is generally poor, and the surgical trauma can intensify the stress response of the body, thus suppressing and weakening the autoimmune ability of the body and affecting the prognosis of patients. The results of this study showed that at 7 days after surgery, the CD4+ and CD4+/CD8+ in both the observation group and the control group were smaller than the preoperative ones in their same groups, and those of the observation group was larger than those of the control group (*P* < .05), which suggested that surgery could suppress the immune function of esophageal cancer patients, but the suppressive effect of endoscope-thoracoscope and laparoscope radical resection on immune function was smaller than that of traditional open surgery. The reasons are considered that endoscope-thoracoscope and laparoscope surgery does not require opening the chest and cutting off the rib cage, which causes less damage to the organism, and the stress reaction is less.^[22]^

The most serious postoperative complication of esophageal cancer is anastomotic fistula, the occurrence of which seriously affects the prognosis and quality of life of patients.^[23]^ Most open esophageal cancer surgeries adopt intrathoracic anastomosis, once an anastomotic fistula occurs, it will easily lead to chest infection and high mortality rate.^[24]^ The main causes of anastomotic fistula are poor anastomotic blood flow, high anastomotic tension, poor anastomotic technique, poor anastomotic approach, cervical incision infection, inadequate postoperative gastrointestinal decompression, and premature feeding. The results of this study showed that, postoperatively, there was no significant difference in the incidence of arrhythmia and anastomotic fistula when compared between these 2 groups, consistent with the results of the D’Amico TA et al^[25]^ study. The occurrence of anastomotic fistula may be mainly related to excessive cropping of the gastric lesser curved side, thus affecting the blood supply to the thoracic stomach.

In terms of respiratory complications, although there was no statistically significant difference in the incidence of complications such as pneumonia, pleural effusion, pneumothorax, and pulmonary embolism between the observation group and the control group (*P* > .05), the incidence was higher in the control group compared to that in the observation group (18.51% vs 8.16%, 20.37% vs 8.16%, 3.70% vs 2.04%, 1.85% vs 0.00%). Pneumonia and pleural effusion were the most significant respiratory complications that differed between these 2 groups. Previous studies have concluded that esophagectomy, especially open esophagectomy, has a higher risk of postoperative respiratory complications.^[26]^ Recent studies have also reported that endoscope-thoracoscope and laparoscope radical esophagectomy reduces these complications.^[26,27]^ This may be due to the traditional thoracotomy, which requires cutting the latissimus dorsi, median abdominal and anterior serratus incisions, but it has a long incision, a large traumatic surface, the need for props to separate the ribs for a longer period of time during surgery, which may cause postoperative patient pain, the severe symptoms of postoperative respiratory impairment, and the postoperative pain that decreases active coughing and coughing, resulting in increased exudate leading to respiratory dysfunction. The results of this study also confirmed this. The reason might be that minimally invasive surgery reduces the disturbance to the heart and lungs, avoids cutting the diaphragm, and maintains the integrity of the thorax and abdomen, alleviates pain, decreases the secretion of various inflammatory factors, and sputum volume is subsequently reduced, and the impact on respiratory function is lightened because the patient is able to cooperate with cough and expectoration due to the smaller trauma.^[28]^ In addition, the 1-year postoperative OS rate was 81.63% (40/49) in the observation group and 72.22% (39/54) in the control group, and the difference was not statistically significant when comparing the OS rates of these 2 groups (*P* = .238, HR = 0.622, 95% CI = 0.279–1.385). It is suggested that both procedures possess equal long-term efficacy.^[29]^

The current study still has some limitations. For example, the endoscope equipment is expensive, the scope of surgical adaptation is narrow, the technical difficulty of the operation is high, the operator requirements are high, and the learning curve is long.^[30]^ This is due to the fact that thoracoscopic surgery is a transition from traditional surgery with a 3D view to a display to a 2D view, and the instruments used to perform endoscopic surgery are different from those used for open surgery. All these factors limit the widespread use of endoscope-thoracoscope and laparoscope radical esophagectomy. Secondly, the sample size included in the current study was small, which may lead to some bias in the study results. Finally, the postoperative follow-up observation period in the current study lasted only 1 year, which is a short follow-up period, and there was no further observation and analysis of the effect of endoscope-thoracoscope and laparoscope radical esophagectomy on the survival rate of patients with esophageal cancer at 5 years after surgery. We need to further expand the sample size and refine the study design to further explore the study results. Additionally, this study is a single-center study, which may introduce local bias. Future research will expand the scope to further validate the findings.

In conclusion, endoscope-thoracoscope and laparoscope radical esophagectomy is a safe treatment option because it causes little trauma to patients with significant recent efficacy. It can effectively protect the immune function of esophageal cancer patients with high safety, which is a safe treatment option.

## Author contributions

**Conceptualization:** Mingquan Ma, Peng Ren, Haitong Wang, Hongdian Zhang, Lei Gong, Yufeng Qiao, Xiangming Liu, Peng Tang.

**Data curation:** Mingquan Ma, Peng Ren, Haitong Wang, Peng Tang.

**Formal analysis:** Mingquan Ma, Peng Ren, Haitong Wang, Lei Gong, Yufeng Qiao, Peng Tang.

**Investigation:** Mingquan Ma, Peng Ren, Haitong Wang, Hongdian Zhang, Lei Gong, Yufeng Qiao, Xiangming Liu, Peng Tang.

**Methodology:** Mingquan Ma, Peng Ren, Haitong Wang, Lei Gong, Yufeng Qiao, Xiangming Liu, Peng Tang.

**Software:** Peng Ren, Xiangming Liu.

**Supervision:** Mingquan Ma, Hongdian Zhang, Peng Tang.

**Validation:** Haitong Wang, Hongdian Zhang, Lei Gong.

**Writing – original draft:** Mingquan Ma, Peng Ren, Yufeng Qiao, Peng Tang.

**Writing – review & editing:** Mingquan Ma, Peng Ren, Yufeng Qiao, Peng Tang.
